# Supplementary material for: Complexome profiling of the Chlamydomonas psb28 mutant reveals TEF5 as an early PSII assembly factor
Source: Plant Cell. 2025 Mar 17;37(6):koaf055. doi: 10.1093/plcell/koaf055 (PMC12164590; doi:10.1093/plcell/koaf055)
Supplement: koaf055_Supplementary_Data [file koaf055_supplementary_data.zip › psb28_interactive.zip!/plots/intensity/qualityPlots/peptides/ORF2971_Cre-1.g2716962.t1.p1.html]

### Cre-1.g2716962.t1.p1

Trivial name: ORF2971  
SeqCoverage [median]: 0.90  
Mass: 341.64 kDa  
  
 

| Sequence | MissedCleavage | Mass [Da] | wtIntensity | muIntensity | Score |
| --- | --- | --- | --- | --- | --- |
| LVANYSK | 0 | 793.43 | 283.85 | 0.00 | 68.92 |
| NQVVYQLTR | 0 | 1119.60 | 0.00 | 294.55 | 66.62 |
| SLNFALISHSK | 0 | 1215.66 | 4530.48 | 392.72 | 72.88 |
|  |  |  | 4814.3 | 687.3 |  |
